# Bottom–Up Metasurfaces for Biotechnological Applications

**DOI:** 10.1002/advs.202413679

**Published:** 2025-02-08

**Authors:** Francesca Petronella, Federica Zaccagnini, Maria Laura Sforza, Vincenzo De Mei, Luciano De Sio

**Affiliations:** ^1^ National Research Council of Italy, Institute of Crystallography CNR‐IC Montelibretti Division Area territoriale di Ricerca di Roma Strada Provinciale 35d Rome n. 9 – 00010 Italy; ^2^ Department of Medico‐Surgical Sciences and Biotechnologies Sapienza University of Rome Corso della Repubblica 79 Latina 04100 Italy

**Keywords:** biosensing, biotechnologies, metasurfaces, optics, pathogens, photo‐thermal, plasmonics

## Abstract

Metasurfaces are the 2D counterparts of metamaterials, and their development is accelerating rapidly in the past years. This progress enables the creation of devices capable of uniquely manipulating light, with applications ranging from optical communications to remote biosensing. Metasurfaces are engineered by rational assembly of subwavelength elements, defined as meta‐atoms, giving rise to unique physical properties arising from the collective behavior of meta‐atoms. These meta‐atoms are typically organized using effective, reproducible, and precise nanofabrication methods that require a lot of effort to achieve scalable and cost‐effective metasurfaces. In contrast, bottom–up methods based on colloidal nanoparticles (NPs) have developed in the last decade as a fascinating alternative for accelerating the technological spread of metasurfaces. The present review takes stock of recent advances in the fabrication and applications of hybrid metasurfaces prepared by bottom‐up methods, resulting in disordered metasurfaces. In particular, metasurfaces prepared with plasmonic NPs are emphasized for their multifold applications, which are discussed from a biotechnology perspective. However, some examples of organized metasurfaces prepared by merging bottom–up and top–down approaches are also described. Finally, leveraging the historical disordered metasurface evolution, the review draws new perspectives for random metasurface design and applications.

## Graphical abstract

Bottom–up metasurfaces represent a new generation of optical components made up of randomly organized meta‐atoms. Due to their exceptional morphological and optical properties, these metasurfaces are utilized in modern applications. These advanced materials have applications in various biotechnological fields, including biosensing and environmental health monitoring.

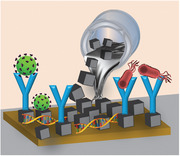

## 1 Introduction

Metamaterials exhibit physical properties that are non‐existent in natural materials. For instance, the electric permittivity (*ε*) and the magnetic permeability (*µ*) of metamaterials can be negative, resulting in a negative refractive index (*n*), which can be exploited for the production of optical devices able to overcome the diffraction limit.^[^ ^1^ ^]^ Metamaterials are obtained by rationally assembly nanostructures in a specific arrangement, according to the desired properties and applications. The nanostructures composing metamaterials are generally known as meta‐atoms. Remarkably, metamaterial properties are not intrinsically dependent on the chemical composition of the physical structure and meta‐atom arrangement. The spatial arrangement of meta‐atoms gives rise to collective properties that dictate the physical properties of the resulting metamaterial, as well as their stimuli‐responsiveness.^[^ ^2^ ^]^ Indeed, the operating wavelengths of metamaterials can be controlled by changing the periodic arrangement of meta‐atoms so that the meta‐atom inter‐distance is shorter than the incident wavelength.^[^ ^3^ ^]^ This condition produces a collective behavior of meta‐atoms underlying the metamaterial macroscopic properties.

However, achieving metamaterials with the desired optical properties is challenging because it requires a 3D organization of the meta‐atoms. To overcome this difficulty, scientists have focused their efforts in the past years on fabricating 2D metamaterials, the so‐called metasurfaces. A metasurface is a 2D analog of a metamaterial. Similar to metamaterials, metasurfaces can control light at the nanoscale, thus representing a “game changer” for the design of novel optical elements. They are 2D metamaterials that, thanks to the in‐plane organization of the meta‐atoms, possess compelling optical properties. Metasurfaces can work in the entire electromagnetic spectrum, and their applications span from programmable on‐demand optics and photonics (e.g., imaging, holography, beam forming) to sensing and light communication.^[^ ^4^, ^5^ ^]^

Metasurfaces can be classified according to several parameters, including their permeability, permittivity properties, operational frequency, applications, periodicity or disorder, active/passive metasurface composition, meta‐atom composition, and fabrication methods. Fabrication techniques can be categorized into lithography‐based and self‐assembly‐based. Lithography‐based methods are top‐down approaches that consist of i) the realization of a mask (reporting the “negative” desired structures), ii) the deposition of a resist on the desired substrate, iii) the application of the mask on the resist layer, iv) the curing step (performed under UV light to transfer the pattern from the mask to the resist), v) the developing steps, required to obtain the designed structure. Lithography‐based methods are extremely precise but require dedicated facilities and high maintenance costs. Moreover, these methods lay down small patterning areas and hinder the production of optical metasurfaces due to the diffraction limit. Consequently, several studies are devoted to developing self‐assembly methods suitable for the mass‐scale production of optical metasurfaces. Most of them require nanofabrication steps; however, they are extremely promising from a technological and environmental standpoint.^[^ ^6^ ^]^

As an alternative, optical metasurfaces can also be fabricated by bottom–up processes that imply the utilization of colloidal nanocrystals.^[^ ^7^ ^]^

In particular, plasmonic NPs, characterized by the localized surface plasmon resonance phenomenon (LSPR), can be arranged in 2D assembly, generating optical metasurfaces with large manufacturing areas. In this case, controlling the plasmonic metasurface interaction with electromagnetic radiation involves the control of the local long‐range coupling among plasmonic NPs. This constraint implies the arrangement of plasmonic NPs in a periodic lattice. Thus, the production of ordered plasmonic metasurfaces is not entirely unencumbered using a template.^[^ ^7^ ^]^

However, the most promising opportunities lie in disordered metasurfaces.^[^ ^8^ ^]^ These metasurfaces, characterized by a random 2D assembly of the meta‐atoms, reveal complex and fascinating optical properties. The balance of these properties with affordable fabrication technologies is a hopeful prospect. A random arrangement of plasmonic NPs offers numerous degrees of freedom, potentially leading to the discovery of new phenomena and the creation of metasurfaces optimized for a wide range of applications.^[^ ^9^, ^10^ ^]^

Designing a disordered metasurface by assembling plasmonic NPs allows, in principle, the building of an optical device meta‐atom by meta‐atom, opening opportunities unthinkable for mainstream fabrication methods. This paper reviews recent advancements in developing and applying disordered hybrid metasurfaces produced through bottom–up processes, including contributions from the authors. Hybrid metasurfaces strategically integrate metallic and dielectric components, leading to enhanced optical phenomena and improved performance compared to purely metallic or dielectric metasurfaces. This approach allows for the amplification of electromagnetic fields, reduced absorption losses, and the creation of innovative material combinations.^[^ ^11^, ^12^ ^]^

Also, for this exotic class of metasurfaces, the particle spacing in these arrays must be comparable to the incident radiation wavelength. In the general scheme, thin metallic layers give rise to the surface plasmon resonances (SPR), arrays of metallic nanostructures result in LSPR, and coupling these effects produces the plasmonic surface lattice resonances (SLRs). These new hybridized photonic‐plasmonic modes exhibit narrow spectral line widths and relatively high‐quality factors (*Q*), indicating the ability to confine light to the fundamental mode. Experiments demonstrated the possibility of exciting SLRs at both normal and oblique light incidence with significantly improved spectral line widths. They show the potential to overcome the intrinsic limitations of conventional nanomaterials, such as weak absorption, strong reflections, and poorly defined spectral features.^[^ ^13^, ^14^, ^15^ ^]^

This article will first discuss effective protocols for fabricating bottom‐up metasurfaces. The second section will summarize recent examples of how disordered hybrid metasurfaces can address biotechnology and biomedical challenges. Due to their unique optical properties, these metasurfaces have significant potential for developing biosensor devices that facilitate label‐free and real‐time human and environmental health monitoring.

## 2 Bottom–Up Methods for Metasurface Fabrication

Bottom‐up methods for metasurface preparation rely on fabricating the nanostructures through the controlled assembly of individual components such as NPs (metallic or dielectric). NPs are utilized as building blocks that engage with one another thanks to short‐range forces such as electrostatic and van der Waals forces. Although less reproducible than the top–down methods, bottom–up techniques benefit from a deep control over physical properties, homogeneity in particle distribution, easy scalability, and the possibility of obtaining a metasurface conformal to the support. For instance, colloidal nanocube‐based metasurfaces can be produced utilizing straightforward solution‐based deposition methods on any surface without limits on the surface dimension and shape.

**Figure 1:**
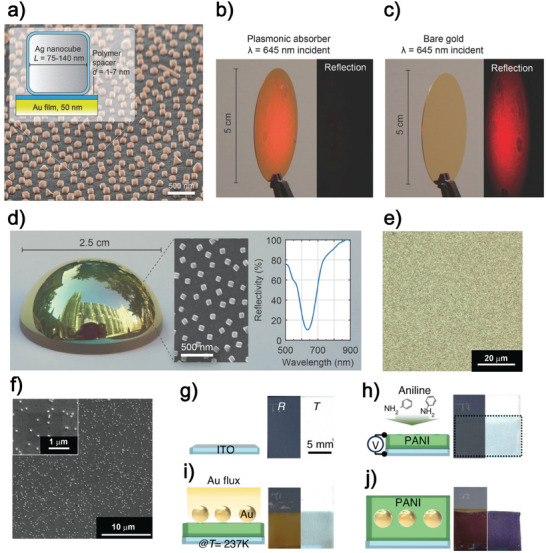
a) Scanning electron microscopy (SEM) micrograph of a flawless absorber surface of AgNCs covering an Au film. The inset shows the cross‐section of a single subwavelength resonator. Images of the Akselrod metasurface (b) and an Au film (c) illuminated by a defocused 645 nm laser. The metasurface shows no reflection (b), while the Au film reflects the laser (c). d) Image of the Akselrod metasurface on a glass half‐sphere, deposited through conformal deposition. The SEM image of the sample's side slope and the near‐normal incidence reflectance spectrum are shown in the insets. Reproduced with permission.^[^ ^16^ ^]^ 2015, WILEY‐VCH Verlag GmbH and Co. KGaA, Weinheim. e) Optical microscopy image of the metasurface reported by Petronella et al., accompanied by the corresponding SEM micrograph (f).^[^ ^17^ ^]^ Reproduced under terms of the CC‐BY license.^[^ ^17^ ^]^ 2023, Petronella et al., published by American Chemical Society. g–j) Fabrication process used by Kim et al. for developing a tunable plasmonic nanofilter.^[^ ^20^ ^]^ The process involves three steps: electrodeposition of PANI on an ITO substrate, physical deposition of thin Au to form AuNPs on PANI, and final electrodeposition of PANI to encapsulate the AuNPs. Reproduced under terms of the CC‐BY license.^[^ ^20^ ^]^ 2024, Gyurin Kim et al., published by Springer Nature.

One of the most relevant examples of metasurfaces fabricated by bottom‐up technique was reported by Mikkelsen and coworkers in 2015.^[^ ^16^ ^]^ Mikkelsen group realized an almost perfect absorber optical component with spectral tunability from the visible to near‐infrared spectrum over sizable regions on conformal surfaces. Colloidal silver nanocubes (AgNCs) were employed as building blocks and deposited on a metal layer using a polyelectrolyte multilayer (PEM) as a dielectric nanoscale spacer (**Figure** **1**a). In detail, the lithography‐free procedure reported by Mikkelsen's group starts with the synthesis of AgNCs, followed by the fabrication of a 50–100 nm thin evaporated gold (Au) layer as a ground plane. After that, a thin dielectric layer (PEM) is assembled on the Au substrate using the electrostatic Layer‐by‐Layer (eLbL) assembly technique. The PEM is a multilayer structure, alternating cationic and anionic polymers, defined as polyelectrolytes (PEs). In the work of Mikkelsen's group, the poly(styrenesulfonate) (PSS) is used as anionic PE, while the poly(allylamine) hydrochloride (PAH) is used as a cationic PE. The PEM exhibits the PAH/PSS/PAH/PSS/PAH sequence. Last, the substrate is exposed to an AgNCs colloidal dispersion, using a 50 µm spacer to distribute AgNCs uniformly. Mikkelsen's group highlighted the versatility of their protocol by demonstrating that the obtained metasurface's morphology, (Figure 1a) can be also achieved on a large area of 5 cm diameter wafer (Figure 1b,c) or a glass half sphere conformal surface (Figure 1d). Inspired by work published by Mikkelsen's group, Petronella et al. fabricated a colloidal metasurface using a slightly modified analogous procedure.^[^ ^17^ ^]^ In this work, AgNCs were self‐assembled and immobilized on a 50 nm thick Au layer using a PEM as a dielectric spacer, giving rise to a 1 cm^2^ area metasurface. The metastructure reported a fill fraction of 4.5% over a 90 µm^2^ surface and an average interparticle distance of 1 µm ± 0.4 µm (Figure 1e,f).

Bottom‐up methods for metasurface fabrication are extremely attractive because of the possibility of easily varying several fabrication parameters (e.g., NP morphology, capping agent, and density thickness of the dielectric layer, reflective layer) to tune the optical characteristics. This issue was addressed by Rozin et al., who systematically investigated the dependence of optical properties on the metasurface morphology.^[^ ^18^, ^19^ ^]^ Rozin's fabrication scheme for colloidal metasurfaces realization involved the use of colloidal nanocrystals of different shapes (nanocubes, spheroids, octahedra) deposited onto an Au film covered with a dielectric spacer. The dielectric spacer was realized using two alternative strategies to tune the thickness. The first approach consisted of passivating the Au film with a monolayer of alkanethiols with a chain length appropriate to obtain a thickness range from 2.9 to 4 nm. Otherwise, the deposition of a uniform layer of poly(methyl methacrylate) (PMMA) by spin‐coating was exploited to obtain a thicker (15–90 nm) dielectric spacer.

Disordered metasurfaces can be fabricated using methods other than self‐assembly. Kim et al. introduced a novel approach to fabricating a disordered plasmonic metasurface. This innovative method involved using conductive polymer nanofilm‐embedded metallic NPs, creating multilayered active plasmonic nanocomposites.^[^ ^20^ ^]^ The authors developed the nanocomposites at the wafer level using a “lithography‐free” technique that involves three consecutive bottom‐up growth processes. As sketched in Figure 1g–j, the polyaniline (PANI), a conductive polymer layer, is electrodeposited on an indium tin oxide (ITO) substrate in the first step. In the second step, AuNPs are produced starting from a thin Au film grown on the underlying PANI layer. Finally, in the third step, further electrodeposition of PANI occurs to encapsulate the AuNPs in a dielectric medium. As reported in the panels of Figure 1g–j, this procedure provides the intended dichroic qualities and sets the basis for the next‐generation color dynamics.

**Figure 2:**
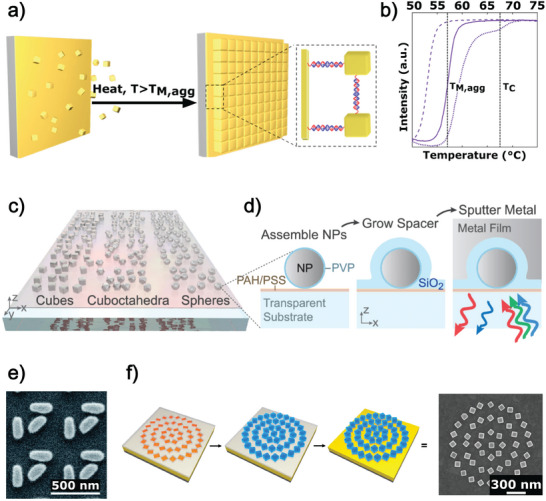
a) Assembly scheme for the large area metasurface investigated by Zheng et al. a,b) creating a 2D crystalline SLs.^[^ ^21^ ^]^ PAE monomers are heated for several hours above the melting point‐temperature of their assembled aggregates (*T*_M,agg_) b) UV–vis melting curves, measured at *λ* = 550 nm, demonstrating temperature‐dependent intensity value. *T*_C_ = 68 °C is the temperature at which there is an equilibrium between crystalline SLs and the melt. The crystallization is facilitated at *T*_C_. Reproduced with permission.^[^ ^21^ ^]^ 2021, American Chemical Society. c) Illustration of several colloidal NPs shapes that self‐assemble on a transparent substrate, d) schematic of the procedure used by Stewart et al. for realizing the self‐assembly of colloidal NPs, with different morphology, on a transparent substrate. Reproduced with permission.^[^ ^22^ ^]^ 2022, American Chemical Society. e) AuNRs trimers patterning composing the flexible chiral metasurfaces produced by H. T. Lin et al.^[^ ^23^ ^]^ Reproduced with permission.^[^ ^23^ ^]^ 2022, The Royal Society of Chemistry. f) Schematic representation of the preparation procedure proposed by Zhou et al.^[^ ^24^ ^]^ to control the orientation and position of prismatic NPs utilizing shallow holes for locally anchoring prismatic NPs, as presented in the respective SEM image. Reproduced with permission.^[^ ^24^ ^]^ 2020, Published under the PNAS license.

Zheng et al. developed a high‐temperature, isothermal growth technique to fabricate crystalline metasurfaces, referred to as crystalline superlattices (SLs), composed of densely packed, DNA‐functionalized AuNCs.^[^ ^21^ ^]^ The method is reported in **Figure** **2**a,b. This fabrication technique utilizes a self‐complementary anisotropic programmable atom equivalents (PAEs) solution. Au‐coated Si wafers are functionalized with thiolated anchor DNA molecules by immersion, and PAEs are obtained from the functionalization of colloidal AuNCs with the same DNA solution. The substrates are then immersed in a PAEs solution and placed on a heated shaker to induce the growth of AuNCs. Finally, samples are brought to room temperature and embedded in an amorphous silica matrix to crystallize. The resulting crystalline metasurfaces SEM images evidence the presence of densely packed AuNCs.

Stewart et al. proposed an original metasurface architecture, summarized in Figure 2c,d. It was obtained by cleverly merging colloidal techniques such as synthesis, self‐assembly, and material growth. Their protocol produced metasurfaces absorbing in a range of 330 to 2740 nm across wafer‐scale areas, as shown in Figure 2d.^[^ ^22^ ^]^ The first step of this procedure is to coat a substrate with a PEM (PAH/PSS) to conformally incorporate plasmonic NPs synthesized in different morphology (spheres, cuboctahedra, or cubes), giving rise to a uniform coating shown in Figure 2c.

Next, using plasma‐enhanced chemical vapor deposition (PECVD), a dielectric coating is grown around the NPs. Finally, a metal layer is physically sputtered around the dielectric‐coated NP (Figure 2d).

As a result, the metasurfaces consist of randomly aligned plasmonic nanocavities supporting an LSPR involving two closely spaced plasmonic surfaces: the assembly of colloidal nanocrystals and the sputtered metal layer. One limitation of bottom–up methods for metasurface fabrication is the inability to achieve a precise NP arrangement with a controlled orientation.

Some fabrication approaches based on combining top‐down and bottom‐up techniques were advanced to address this challenge. Indeed, bottom–up approaches can produce chiral metasurfaces supported by lithographic techniques. H. T. Lin et al. realized a flexible chiral metasurface composed of plasmonic nanorod trimers (Figure 2e) deposited on a polydimethylsiloxane (PDMS) substrate through a simple bonding method, enabling the fine‐tuning of its circular dichroism (CD).^[^ ^23^ ^]^ The realization of such a device involves multiple steps. First, the metasurface patterns were defined through electron beam lithography (EBL) on indium phosphide substrate followed by Au deposition and lift‐off process. To improve the adhesion of Au with PDMS, the device was treated with MPTMS (3‐mercaptopropyl trimethoxysilane) and flipped upside down on the prepared PDMS mixture. The indium phosphite substrate was removed with a hydrochloric acid solution after curing PDMS. The strong covalent bond between the treated Au and PDMS enabled the transfer of the metasurface pattern on the PDMS substrate.

Zhou et al. also exploited the strategic use of lithography techniques. The innovative combination of lithography and bottom‐up method leads to the precise control of the position and orientation of NPs, opening up new possibilities in metasurface fabrication.^[^ ^24^ ^]^

The preparation method is a shallow‐template‐assisted, DNA‐mediated assembly technique. They demonstrated that almost any prismatic‐shaped anisotropic NP may be precisely positioned and oriented on the desired reflective surface in a specific configuration.

The shallow‐template‐assisted DNA‐mediated assembly technique involves the fabrication of lithographically defined pores using a top‐down approach, followed by the assembly of NPs. Initially, a PMMA layer is deposited onto the Au substrate through spin coating. Next, pores with an appropriate aspect ratio (length and thickness) are created using EBL. These pores are then functionalized with a DNA oligonucleotide, facilitating the controlled and localized assembly of cubic NPs that have been functionalized with the complementary DNA sequence. The assembly of DNA‐functionalized NCs on binding sites hosted in the pores occurs under precise shaking conditions (1000 rpm). Complementary DNA‐functionalized nanocrystals are permitted to assemble onto specific binding sites of substrates. Usually, the assembling procedure takes less than two hours to complete. Following assembly, the substrates are washed three times with a 0.5 m sodium chloride buffer solution to remove the unbounded NPs, and then the PMMA template is removed by a suitable washing procedure. After removing PMMA, the substrates were blown dry with nitrogen and washed thrice with an 80% isopropyl alcohol and 0.2 m ammonium acetate solution. As reported in the SEM image of Figure 2f, the resulting metasurface is characterized by a controlled orientation and position of NCs.

The precise NPs arrangement realized by Zhou et al. was achieved by fabricating hollow and anisotropic pores in the PMMA sacrificial template. Indeed, the authors claimed that if the pores present a high aspect ratio and, in particular, a thickness lower than the NP's hydrodynamic radius, the diffusion barrier is reduced, and the incorporation of the DNA functionalized Au layer is promoted. Indeed, the statistical analysis on the NPs offset indicates that the lateral offset is reduced for shallow squared pores, compared to deep pores. Consequently, shallow pores enable precise tailoring of NPs' position and orientation.

### 2.1 Optical Properties of Bottom–Up Metasurfaces

As discussed in Section 2, the interest devoted to bottom‐up metasurfaces is driven by the possibility of achieving metasurfaces with different optical properties by manipulating several parameters involved in the preparation processes. In 2015, Mikkelsen and coworkers demonstrated the ability to control the optical properties of metasurfaces by varying the size of AgNCs and the thickness of the nanoscale spacers. Each AgNC, coupled to the metal layer through the dielectric spacer, works similarly to a magnetic dipole. In particular, the numerous AgNCs acting collectively on the surface produce an efficient magnetic response, ultimately resulting in impedance (*n*) matching. This collective behavior is responsible for perfect absorption as it eliminates the surface's reflection and transmission, matching the surface's impedance to the surrounding medium (air). The formation and decay of surface plasmons in the space between the top of the metal film and the bottom AgNC facet dissipates the incident light.^[^ ^16^ ^]^ The resulting metasurface's optical properties are reported in the inset of Figure 1d. The surface's reflectance spectrum shows a 90% absorption at the resonant wavelength (*λ* = 635 nm). Interestingly, the authors demonstrated that it is possible to modify the resonance wavelength and the percentage of reflectivity by varying the incubation time and the AgNCs density so that the absorption can theoretically approach the virtually perfect absorption behavior. Figures 1b,c show that the metasurface exhibits minimal reflection under resonant illumination at *λ* = 645 nm. The residual scattering from the larger noncubic particles and NCs gives the coated wafer a red appearance (Figure 1b). In contrast, significant light reflection is observed when a 645 nm laser hits a conventional Au film.

**Figure 3:**
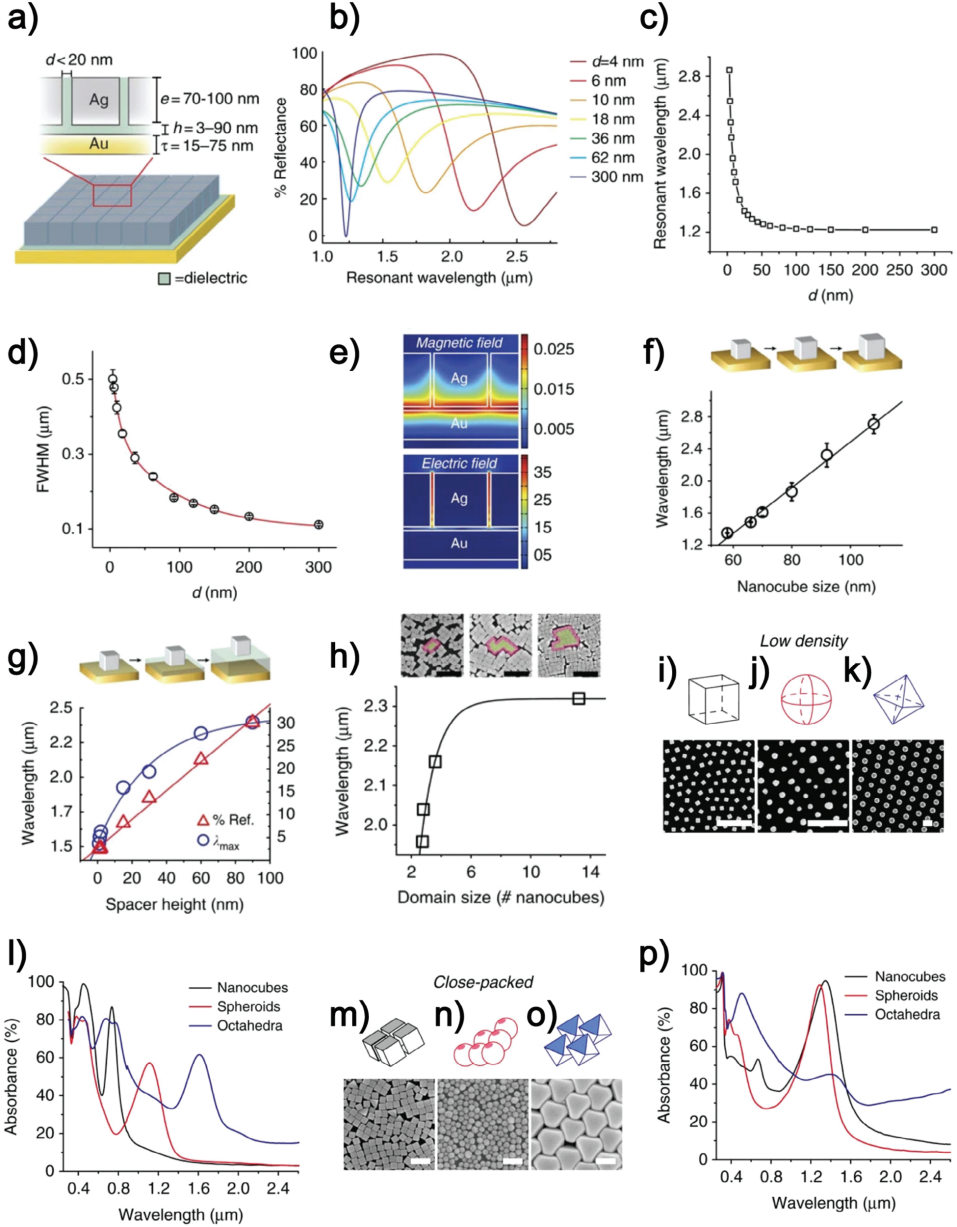
a) Schematic of the metasurface studied by Rozin et al.^[^ ^18^ ^]^ b) Simulated reflectance spectra of Rozin's metasurface calculated for different interparticle distances. c) Fundamental resonance wavelength variation as a function of interparticle distance. d) Variation of the FWHM as a function of interparticle distance. e) Calculated magnetic and electric field intensities for a close‐packed metasurface (*d* = 4 nm) at the fundamental resonance wavelength (*λ* = 2.54 µm). f,g) Variation of the fundamental resonance wavelength as a function of nanocube size (f) and spacer height (g), obtained via 2D FDTD simulations. h) Fundamental resonance wavelength variation for metasurfaces with domains of meta‐atoms (AgNCs) of different sizes, along with corresponding SEM micrographs (scale bar: 500 nm). i–l) SEM micrographs (scale bar: 500 nm) of low‐density metasurfaces using NCs (i), spheroids (j), and octahedra (k) as meta‐atoms, demonstrating shape‐dependent optical properties (l). m–p) SEM micrographs (scale bar: 500 nm) of close‐packed metasurfaces using NCs (m), spheroids (n), and octahedra (o) as meta‐atoms, showing shape‐dependent optical properties (p). Reproduced under terms of the CC‐BY license.^[^ ^18^ ^]^ 2015, Matthew J. Rozin et al., published by Springer Nature.

Analogous results were obtained by Rozin et al. The authors proved the possibility of tuning the optical characteristics of a colloidal metasurface by varying some of its fabrication parameters.^[^ ^18^ ^]^ **Figure** **3**a–p summarizes these results. The optical response of Rozin's metasurface varies depending on several parameters, including the length of the nanocube edge (e), the interparticle distance (*d*), the dielectric spacer height (*h*), and the thickness of Au film (*t*). The possibility of obtaining a near‐ideal electromagnetic absorbance that is tunable from the visible to the mid‐IR is demonstrated. First, using 2D finite‐difference time‐domain (FDTD) simulations, the optical response of metasurfaces made of 92 nm AgNCs of different interparticle spacings is predicted. Simulations were performed considering interparticle spacings spanning 3 to 300 nm (Figure 3a–e). Figure 3c,d show that the absorption peak's position and linewidth decrease when d increases This simulated trend has a theoretical explanation: the larger the interparticle spacings are, the weaker the coupling between the NPs, and the optical resonance approximates that of a single isolated NP when the meta‐atoms are closed‐packed; instead, the out‐of‐plane coupling between the metal film and LSPR, which is approximately continuous, results in the broadening of the absorption peak (caused by radiation damping) and in its redshift. Figure 3f–h shows the possibility of tuning the metasurface optical properties by varying the nanocube size, the dielectric spacer height, and the nanocube packing density. Increasing the AgNC size not only increases the wavelength of the absorption peak but also decreases the absorption efficiency of the fundamental mode. This decrease is expected to result from the enhanced scattering cross‐section of larger NPs. Such behavior affects the quality factor for the fundamental gap mode, Q. This figure of merit was purposely introduced to compare the effects on the efficiency of the resonator induced by the nanoparticle shape alone. Q is defined as follows:

(1) $$Q=\lambda_{0}/\delta \lambda$$

where *λ*_0_ is the peak wavelength, and *δλ* is the resonant peak full width at half maximum (FWHM).

In closely packed colloidal metasurface samples, increasing the height of the dielectric spacer results in a redshift of the resonant wavelength (see Figure 3g). A thicker spacer weakens the out‐of‐plane field confinement between the Au substrate and the NPs while enhancing the in‐plane field confinement among the NPs. In their work, Rozin et al. observed that the meta‐atoms can generate domains of different sizes where AgNCs are close‐packed according to the fabrication conditions. The domain size affects the optical properties of the resulting metasurfaces: the absorption wavelength redshifts with the increase of the domain size (Figure 3h). Moreover, optical absorbance at the resonance becomes more intense when the domain size is larger. Therefore, the metasurface's optical response becomes more and more similar to that of a perfect array as the meta‐atom domains become more ordered. The dimensions, size distribution, and meta‐atom surface chemistry are the main parameters dictating the average interparticle distance and domain sizes. For disordered metasurfaces, Rozin et al. investigated the effect of meta‐atom shapes on the Q and the wavelength of the fundamental resonance both for low‐density and close‐packed metasurfaces (Figure 3i–p). For low‐density metasurfaces (Figure 3i–l), the meta‐atoms shape affects the Q according to the equation:

(2) $$\left(Q_{\mathrm{sphere}}=3.8\right)<\left(Q_{\mathrm{octa}}=6.1\right)<\left(Q_{\mathrm{cube}}=7.2\right)$$

This relation is a direct consequence of the nanocrystal's different shapes. NCs form a well‐defined parallel cavity with the metal substrate, and these cavities, thanks to low‐loss confinement, give rise to high‐quality absorption resonances. The quality factor associated with NCs is 7.2. Octahedra *Q* value, instead, is 6.1, smaller than one of the cubes because a non‐negligible part of this NP extends out of the cavity. Spheres, finally, are associated with poor field confinement due to their high curvature. Consequently, spheres have the lowest Q of 3.8. For close‐packed metasurfaces (Figure 3m–p), the strong coupling of metaatoms with the underlying Au significantly redshifted the absorption peak to the one associated with low‐density configurations in the case of NCs and octahedra. Conversely, the redshift is not observed for spheroids (Figure 3p) since they exhibit a weak interaction between the in‐plane and the out‐of‐plane gap modes. This study underlines the non‐negligible role of the resonator shape choice in tuning a colloidal metasurface resonance. The fundamental peak position and FWHM also depend on the nanocrystal's chemical composition since each element corresponds to specific plasmonic properties.^[^ ^19^ ^]^ As a result, AgNCs are often selected as ideal candidates for constructing well‐defined parallel cavities with the metal (Au) substrate in metasurfaces.

Instead, the thickness of the Au film, as well as the interparticle distance and the thickness of the dielectric layer (both changed as a function of the DNA length), were used by Zheng et al. to tune the resonant wavelength of the metasurface shown in Figure 2a in the near‐IR range.^[^ ^21^ ^]^

**Figure 4:**
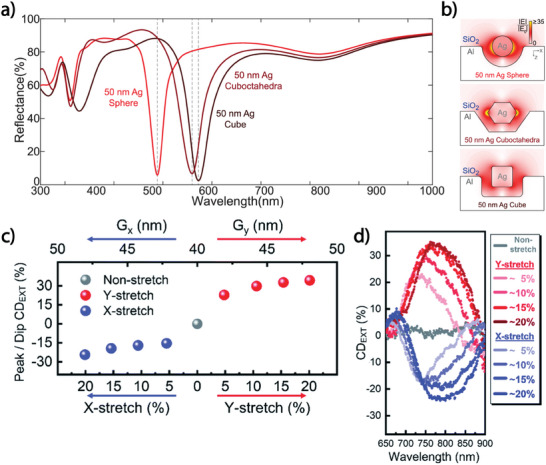
a) Simulated reflection spectra for three metasurfaces made up of the three NP morphologies displayed in Figure 2e. They demonstrate how the plasmonic cavity's geometry affects the wavelength of resonant absorption. b) Electric field enhancement of these geometries at the fundamental plasmon resonance. Reproduced with permission.^[^ ^22^ ^]^ 2022, American Chemical Society. c) CD magnitude variation obtained by the controlled stretching of the substrate realized by H. T. Lin et al.^[^ ^23^ ^]^ d) CD extinction stretching the substrate along the *y* and *x*‐axes. Reproduced with permission.^[^ ^23^ ^]^ 2022, The Royal Society of Chemistry.

Also, in the work by Stewart et al., the plasmon resonance is regulated by adjusting the nanostructures' size, material, and geometry.^[^ ^22^ ^]^ The proposed arrangement of NPs and dielectric/metallic coatings enabled the excitation of the plasmonic resonances between NP and sputtered metal films. Such an approach prepared a metasurface with NPs with three geometries: AgNCs, Ag cuboctahedra, and Ag spheres. Numerical simulations show how the NP's geometry impacts the reflectance properties of the resulting metasurface (**Figure** **4**a). Indeed, the maximal absorption at the fundamental plasmon resonance occurs at 575 nm for the cubic NPs, 490 nm for the spherical NPs, and 560 nm for the cuboctahedral NPs. Figure 4b shows the electric field enhancement of these geometries at the fundamental plasmon resonance. Figure 4b evidences that the bulk of the electric field is concentrated within and close to the NP surfaces. This NP‐only plasmon oscillation is connected to the opposite phase oscillation in the sputtered metal film. Metasurfaces consisting of these elements absorb a substantial amount of energy because the electric field in the far field is reduced or canceled by the coupling modes between the NP and the film, which prevents the incident energy from being reradiated.^[^ ^22^ ^]^

H. T. Lin et al. used the strategy of varying the strength and phase of the asymmetric coupling between LSP waves around the metallic nanorods (NRs) arranged as trimers (Figure 2e) to induce optical chirality. The optical chirality is induced by stretching the flexible device.^[^ ^23^ ^]^

The asymmetric couplings between the center and the side of NRs were required features for producing optical chirality and corresponded to different inclinations of left and right circularly polarized light. The magnitude of the CD extinction spectrum increased with higher gap distances in the NR trimers. The device stretching enables the fine‐tuning of LSPR couplings and the manipulation of the circular polarization degree of the incident light extinction with a broad tuning range since NR trimers become more asymmetric under stretching, leading to variable levels of chirality. Different stretch scenarios were analyzed, collecting the CD extinction (Figure 4c). However, its variation is not linear with the PDMS strain on the nanometer scale, and the redshifts of the spectra demonstrated an increase in the period of NRs during the stretching. The variation of the peaks in the spectra versus the amount of stretch applied shows that for the stretching along the *y‐*axis, the CD extinction is positive, indicating a right‐ended circular polarization. In contrast, when stretching is along the *x*‐axis, the CD extinction is negative, indicating left‐handed circular polarization (see Figure 4d).

By changing the level and direction of the stretching, the active tuning range of 55% in CD extinction was achieved. The durability and reliability of the flexible chiral metasurface were validated.

In summary, the unique optical properties of bottom‐up metasurfaces arise from the in‐plane and out‐of‐plane coupling of the plasmonic nanostructures used as a building block. Consequently, to access the desired optical properties, the arrangement of plasmonic nanostructures and the thickness of the reflective and dielectric layers are extensively explored. This approach, although effective, provides only a passive tuning of the optical properties. Conversely, a key breakthrough in unlocking the application potential of bottom–up metasurfaces lies in their ability to modulate their optical properties dynamically. Two recent research papers demonstrated this possibility. In the first, the metasurface optical properties were actively changed by dynamically varying the *n* of the surrounding medium.

**Figure 5:**
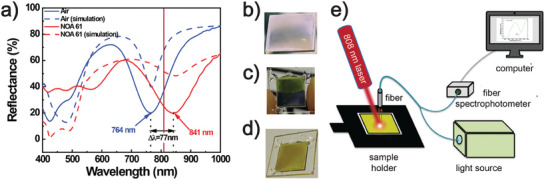
a) Theoretical and experimental study on the sensitivity of the metasurface to *n* changes, as shown by the image of the metasurface (b) and the metasurface cell during infiltration with NOA‐61 (c), which demonstrates a significant color change (d). e) Schematic illustration of the adopted customized spectroscopic‐photo‐thermal apparatus.^[^ ^17^ ^]^ Reproduced under terms of the CC‐BY license.^[^ ^17^ ^]^ 2023, Petronella et al., published by American Chemical Society.

In particular, the bottom‐up metasurface prepared and characterized by Petronella et al. (Figure 1e,f) exhibits a reflectance dip at 764 nm and a reflection efficiency of 60% (**Figure** **5**a). The authors investigated theoretically and experimentally the optical properties of the resulting system, demonstrating high sensitivity to n change (Figure 5a) and light‐to‐heat conversion ability.

In particular, the metasurface sample, infiltrated with a high n material (NOA‐61), displayed a redshift of the reflectance dip wavelength and, consequently, a vivid color change (Figure 5b–d).^[^ ^17^ ^]^

The light‐to‐heat conversion ability was examined under NIR laser irradiation using the customized photo‐thermal setup sketched in Figure 5e. As the sample, under 808 nm irradiation, reached a temperature above 70 °C, the metasurface photo‐thermal properties were exploited to realize a thermoplasmonic‐controlled optical absorber. To this end, the authors ingeniously combined the large area metasurface with a planarly aligned nematic liquid crystal (NLC) layer. Such a strategy allows for the photo‐thermal tuning of the position of the reflectance dip of the metasurface over 46 nm using an intensity value of 8.4 W cm^−2^, thus resulting in a light‐controllable optical device.

The second example is a metasurface fabricated by Kim et al. It exhibits three major optical modes, accompanied by scattering processes that result in different dichroic reflection and transmission colors. A critical accomplishment in this case is that every hue in the visible spectrum may be electronically altered by applying an external voltage of less than 1 V and a switching time of 3.5 s. Additionally, the color temperature of white light may be effectively and dynamically modulated across the warm‐to‐cool spectrum (3250–6250 K) thanks to its electrically programmable multicolor capability.^[^ ^20^ ^]^

## 3 Environmental Application of Bottom–Up Metasurfaces

Hybrid disordered metasurfaces currently find their main applications in domains that exploit their outstanding *n‐*change sensitivity and photo‐thermal properties. For this reason, several efforts are devoted to developing metasurface‐based biosensors. Biosensors use biological molecules and biochemical reactions to detect chemical/biological compounds by electrical, thermal, piezoelectric, or optical signals.^[^ ^25^ ^]^ They rapidly and less expensively detect pathogens, nucleic acids, enzymes, proteins, and other biological compounds.^[^ ^26^ ^]^

Metasurfaces are ideal candidates as transducers for developing biosensors in environmental and healthcare contexts with different readout methods. The following sections will delve into compelling examples of bottom–up metasurfaces used to detect pathogens or molecules with environmental relevance and diagnostic interest, particularly in healthcare. Moreover, the versatility of disordered metasurfaces in fabricating biosensors on flexible supports opens up a wide range of potential applications, inspiring new avenues of research and development.

Hybrid metasurfaces with randomly oriented metallic NPs have been successfully used to detect several pathogen strains via antigen‐antibody interactions, resulting in immunosensors. Our group exploited antibody‐functionalized AuNRs (Ab‐AuNRs) deposited on a PEM‐coated glass to detect *Escherichia coli (E. coli)* in potable water.^[^ ^27^ ^]^

The deposition of PEM and AuNRs occurred via the immersive eLbL assembly, and LSPR was exploited as the readout technique. Indeed, the wavelength of the LSPR absorption signals depends on the *n* of the medium interacting with plasmonic NPs. Consequently, a *n* change, caused, for example, by environmental contamination or the presence of a molecule, causes a variation in the resonance frequency (LSPR peak wavelength). The change in frequency manifests itself with a shift in the absorption bands, proportional to *n* variation and consequently proportional to the quantity of the molecule that has altered the medium. This phenomenon is thus exploited to develop optical biosensors.^[^ ^28^ ^]^

**Figure 6:**
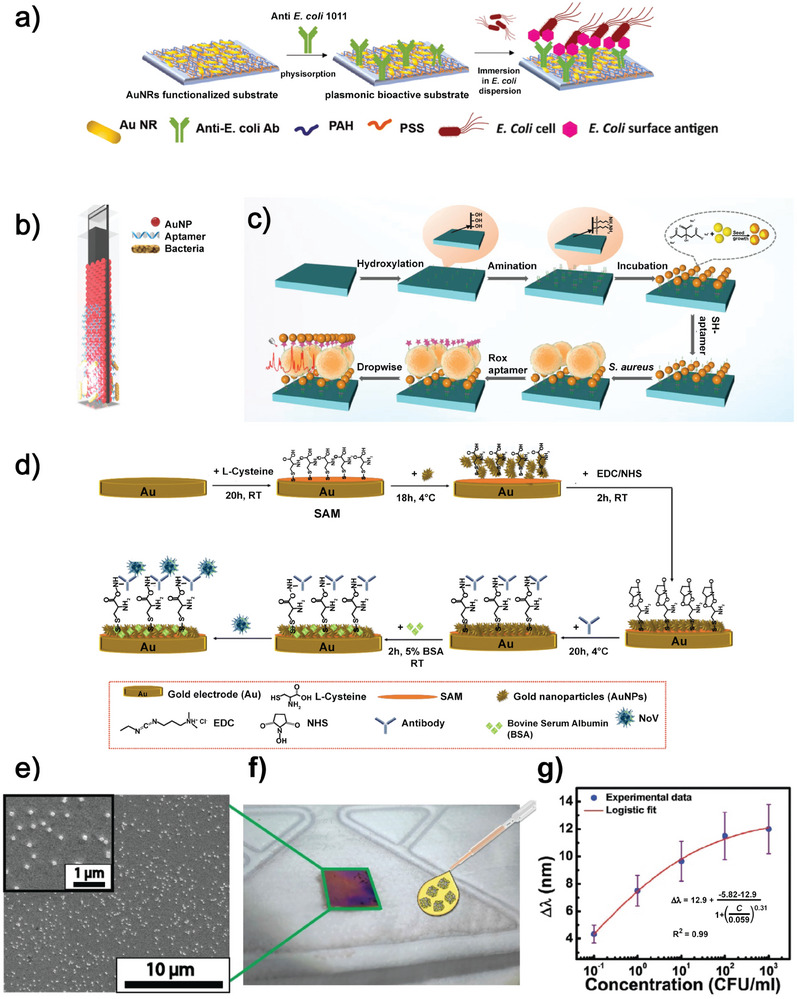
a) AuNRs immobilized on a PEM‐coated glass substrate. The metasurface was functionalized with an antibody for *E. coli* detection. Reproduced with permission.^[^ ^27^ ^]^ 2022, The Royal Society of Chemistry. b) AuNPs on APTES‐coated glass. Functionalization occurred with an aptamer to detect *S. typhimurium*. Reproduced with permission.^[^ ^32^ ^]^ 2017, Springer Nature c) AuNPs and AgNPs on APTES coated PDMS substrate. Functionalization occurred with an aptamer to detect *S. aureus*. ROX and another layer of AuNPs and AgNPs were used as Raman reporters. Reproduced with permission.^[^ ^33^ ^]^ 2023, Elsevier B.V. d) AuNPs and Au electrodes separated by *L*‐cysteine layer to detect Norovirus. Reproduced with permission.^[^ ^36^ ^]^ 2024, The Royal Society of Chemistry. e) SEM image of the optical metasurface integrated into the multifunctional face mask. f) Photo of the optical metasurface biosensor integrated on the FFP2 face mask functionalized surface. g) Calibration curve of the *E. coli* qualitative and quantitative detection. Reproduced with permission.^[^ ^37^ ^]^ 2024, Wiley‐VCH GmbH.

Once bioactivated with a monoclonal antibody raised against *E. coli* cells, this metasurface can be used as an optical transducer able to detect the target microorganism dispersed in water by absorption spectroscopy, with a detection limit of 8.4 CFU mL^−1^ (**Figure** **6**a).

Interestingly, the metasurface obtained by Petronella et al. is reusable since, thanks to the thermoplasmonic properties of the AuNRs, it can be disinfected by NIR‐laser illumination.^[^ ^27^ ^]^

Furthermore, the metasurface was optimized in its optical and morphological characteristics to effectively achieve photo‐thermal disinfection of medical tools contaminated with *E. coli*. This process can now be carried out using simple white light, making it a more cost‐effective and safer alternative to laser illumination.^[^ ^29^ ^]^

Such a goal, supported by numerical simulation, leverages the unique thermoplasmonic properties of noble metal NPs. Indeed, they can generate heat locally if a light source close to the plasmon resonance frequency illuminates the NPs. The light is absorbed, exciting the oscillating plasmons, causing a non‐equilibrium condition and energy dissipation via heat.^[^ ^30^ ^]^

In addition, it was successfully demonstrated that by combining the optimized metasurface with light‐responsive NLC in a cascade‐like arrangement, it is possible to detect bacteria in a wide concentration range from 10 to 10^9^ CFU mL^−1^, lowering the limit of detection (LOD) for *E. coli* from 8 to 1 CFU mL^−1^.^[^ ^27^, ^31^ ^]^ NLCs modified with light‐responsive molecules (azobenzene) were, in fact, more sensitive in detecting high concentrations of bacteria, compared to LSPR, susceptible to saturation in the presence of high concentrations of target cells.

Another method for detecting pathogens is to use bacterium‐specific aptamers as a biorecognition element. After preparing a metasurface by depositing AuNPs onto a (3‐Aminopropyl)triethoxysilane (APTES)‐coated glass substrate (dielectric) via amine‐linking, Oh et al. functionalized the AuNPs with a thiolated aptamer for *Salmonella typhimurium (S. typhimurium)*. Again, contamination occurred using various concentrations of bacteria from cultures and contaminated food. Recognition of *S. typhimurium* occurred via LSPR, but, in this case, the alteration of the medium caused by bacteria had an impact on the absorption intensity and not on the position of the plasmon band (Figure 6b).^[^ ^32^ ^]^ Zhu et al. deposited a mix of AuNPs and AgNPs, functionalized with the aptamer for *Staphylococcus aureus (S. aureus)*, on a PDMS substrate. After contamination with bacteria via infected water, food, and milk samples, further functionalization was carried out with a 6‐carboxy‐*x*‐rhodamine (ROX) aptamer and an additional layer of Au‐AgNPs (Figure 6c). These last two layers served as Raman reporters and hot spots, allowing surface‐enhanced Raman scattering (SERS) to recognize *S. aureus*.^[^ ^33^ ^]^

When a photon is absorbed, it interacts with the vibrational modes of a molecule and is re‐emitted with a different frequency, resulting in the Raman scattering signal. Raman scattering is, therefore, inelastic scattering and has a low probability of occurring, making its weak signal of limited use without appropriate amplification. A significant enhancement of the Raman signals is obtained thanks to the electric field of plasmonic nanostructures such as Au or Ag. SERS is extensively exploited for biosensing because it allows the scattering signal to be precisely and unequivocally associated with the composition of the analyte.^[^ ^34^ ^]^

Zhou et al. also employed SERS and DNA hybridization to detect DNA associated with transgenic maize. Au nanoflowers (AuNFs) were deposited on an APTES‐coated silicon (Si) wafer and functionalized with a biotin‐hairpin DNA (Bio‐H1). Hybridization between Bio‐H1 and the target DNA caused the opening of the hairpin and hybridization with an additional biotin‐hairpin DNA (Bio‐H2), thus generating a hybridization chain reaction (HCR) amplification. Bio‐H2 was, in turn, linked via biotin‐streptavidin binding to Ag‐AuNRs tagged with biotin‐DNA‐cyanine 5 as Raman reporters to create a strong enough signal and detect the target DNA via SERS.^[^ ^35^ ^]^

Disordered hybrid metasurfaces have been reported to be extremely useful and versatile in the field of biosensing. They enable the creation of devices that can be tailored to specific needs. Figure 6 provides an overview of some of the configurations discussed in this section.

In addition to bacteria, viruses could also be detected using hybrid disordered metasurfaces. For the recognition of Norovirus in food, Janicka et al. realized a metasurface using an Au electrode and AuNPs interspersed with a monolayer of *L*‐Cysteine amino acid, as a dielectric medium, thus obtaining an electrochemical (EC) sensor.^[^ ^36^ ^]^ In particular, the Au electrode was incubated first in *L*‐Cysteine, then in the AuNPs dispersions, and, subsequently, the system was functionalized with antibodies. The obtained metasurface was contaminated with Norovirus at various concentrations, and food samples were contaminated by the virus, as represented in Figure 6d. The presence of the virus was detected using differential pulse voltammetry (DPV). Once bound to the antibody, the virus increased electric resistance, causing the current to decrease proportionally to its concentration. Recently, we have employed the metasurface reported in^[^ ^17^ ^]^ for biosensing applications.^[^ ^37^ ^]^ The metasurface was integrated into the FFP2 facemask, thus obtaining a biosensing system that can monitor the eventual facemask contamination and, therefore, the safety of a personal protective device (Figure 6e,f). First, the bare metasurface was immersed for 4 hours in a solution containing the anti‐E. *coli* antibody. Next, it was washed by immersion in Milli‐Q water, dried, and integrated into the facemask. At this stage, *E. coli* (model pathogen) dispersions having different concentrations were sprayed on the face mask to simulate a form of pathogen contamination (droplet effect). The optical response of the metasurfaces was assessed by measuring the reflectance spectra after the contamination. The spectra indicate that the contact between *E. coli* cells and the metasurface determined a redshift of the reflection band. It follows that the antibody promoted the capturing of the *E. coli* cells on the metasurface, also demonstrated by contrast phase microscopy. Consequently, the redshift of the reflectance band indicates, spectroscopically, the contamination from the target pathogen. It is relevant to observe that the redshift values of the metasurface reflectance wavelength increased as the *E. coli* concentration increased. Indeed, the metasurface redshift (Δ*λ*) is reported as a function of *E. coli* concentration. Experimental points were interpolated with a four‐parameter logistic function. The behavior of the metasurface as an *E. coli* biosensor pointed out its ability to spectroscopically detect extremely low bacteria concentrations, having a LOD of 1 CFU 100^−1^ mL^−1^. The versatility and specificity of the metasurface biosensor are demonstrated. (Figure 6g)

Bioaerosols (airborne particulate matter of biological origin) are relevant targets to detect as they are a form of outdoor and indoor air pollution that affects human health.^[^ ^38^ ^]^ Thus, innovative techniques must be implemented as alternatives with respect to polymerase chain reaction, PCR. Qiu et al. developed an LSPR sensor chip with annealed Au nanoislands (AuNIs) prepared by thermal dewetting. This chip was used for fast and quantitative detection of total bioaerosol concentration. The LOD for model bacteria *E. coli* and *Bacillus subtili*s were 0.5119 and 1.69 cells mL^−1^. In the process, thiolate ligands were immobilized on AuNIs, followed by the activation of carboxyl groups. This step is crucial in the development of the sensor. The interferometric sensing system was then applied to measure the plasmonic phase changes, providing high sensitivity for bioaerosol detection. Succinimidyl‐ester‐functionalized‐AuNIs can detect the changes in the *n* through binding events. Since bioaerosols cause a more significant *n* change than non‐biological NHx, the plasmonic sensor is adapted to bioaerosol detection in standard living environments, while aggressive environments require further support.^[^ ^39^ ^]^

## 4 Healthcare Applications of Optical Metasurfaces

Hybrid metasurfaces offer significant opportunities as transducers in different spectral ranges for developing biosensors for human health monitoring. These engineered surfaces can detect various biomarkers, such as glucose, proteins, enzymes, nucleic acids, and tumor biomarkers.

Hybrid metasurfaces, with their unique combination of materials, provide sensitivity, selectivity, and real‐time response. This makes them ideal for compact or wearable devices and particularly suitable for continuous monitoring, a key feature in healthcare technology. The following section will provide an overview of some relevant examples of hybrid metasurfaces prepared by bottom‐up‐based approaches or lithography‐free methods specifically designed as biosensors in healthcare applications.

**Figure 7:**
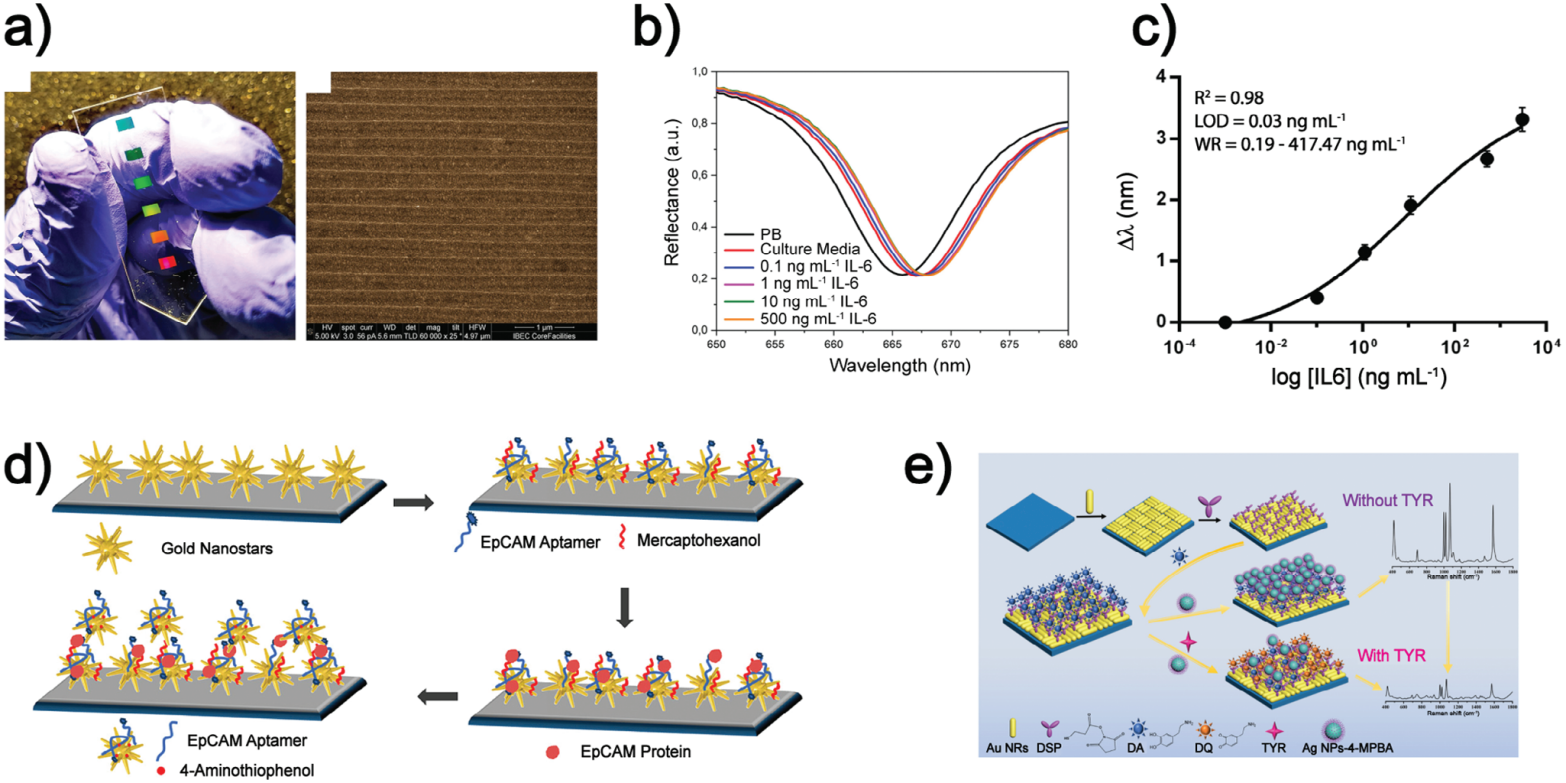
Optical metasurfaces for biomedical applications. a) Photo highlighting the peculiar diffraction of light of the 70 nm Au evaporated Blu‐Ray disc substrate (on the left) and SEM micrograph of the plasmonic nanocrystals (on the right). b) Reflectance spectra obtained at different concentrations of spiked cell culture showing the plasmonic band displacement. c) Calibration curve of the IL‐6 detection.^[^ ^40^ ^]^ Reproduced under terms of the CC‐BY license.^[^ ^40^ ^]^ 2021, The Authors, published by De Gruyter. d) Schematic representation of the procedure for the substrate to capture epithelial cell adhesion molecule protein. Reproduced with permission.^[^ ^44^ ^]^ 2018, American Chemical Society. e) Schematic representation of the specific process of this detection strategy, detecting TYR activity. Reproduced with permission.^[^ ^42^ ^]^ 2022, American Chemical Society.

G. A. Lopez‐Munos et al. realized a label‐free sensor made of nanostructured polycarbonate substrates.^[^ ^40^ ^]^ After eliminating the adhesive layer, a 70 nm thick layer of Au was deposited by resistive thermal evaporation on a large‐area nanostructured array in Blu‐ray discs. (**Figure** **7**a). The metallic thickness and the angle of incidence of light for reflection detection were selected to evaluate the optimal response of the antibody‐antigen detection while performing FDTD numerical simulations. The authors demonstrated that 70 nm thickness enables the maximum plasmon‐exciton coupling and surface sensitivity. The incident angle was analyzed with the addition of 10 nm of biolayer. The Bloch wave surface plasmon polariton mathematical model describes the plasmonic band behavior: higher incident angles displace the plasmon band toward the NIR range. The higher surface sensitivity was found at 40° since the bulk sensitivity increases for higher incident angles while the surface sensitivity decreases. Then, the plasmonic substrate was activated with a self‐assembled layer of carboxylic acid to bind the IL‐6 antibody easily through highly stable amine bonds. Different concentrations of IL‐6 (a cytokine involved in several inflammatory processes) were detected with an incident angle of light of 40°, and the respective plasmonic band displacements were observed in Figure 7b. The LOD of 0.03 ng mL^−1^ was calculated from the calibration curve in Figure 7c, exceeding the sensitivity of nanoplasmonic refractometric biosensing and reaching similar values of the more complex and labeled secondary antibody sandwich essay.

Metasurfaces can serve as optical transducers in biosensing devices that monitor the phenomenon of CD. Biomolecules, such as proteins, possess a distinct chirality that is closely linked to their functionality. Therefore, detecting a biomolecule alongside its chirality is a significant concern in biomedical applications. CD is a spectroscopic technique that enables the recognition of enantiomers. Conversely, terahertz (THz) absorption spectra provide unique spectroscopic fingerprints for identifying specific biomolecules. However, one limitation of THz spectroscopy is its inability to differentiate between enantiomers. By combining THz absorption with CD, it is possible to identify a particular biomolecule and its chirality. This objective can be accomplished using chiral metasurfaces, which create a superchiral field that enhances the CD signal of a substance. With this in mind, Z. Wang et al. realized a terahertz chiral metasurface to provide fast and low‐cost detection of chiral compounds.^[^ ^41^ ^]^ The construction process involves two steps. The aluminum substrate is selected as a reflective substrate. Then, a Kapton commercial polyimide film tape is added to the substrate as a carbon precursor to generate the bottom laser‐induced graphene antenna. The graphene antenna is produced once the first laser‐induced by the writing process is conducted. Upon the antenna stacked on the substrate, a second polyimide film is added. A second laser direct writing step generates the top antenna layer. The CD spectra highly depend on the twisting angle; thus, a strict alignment of the two antennas is crucial for the measurements. A twisting angle of 45° is selected to achieve maximum signal and greatest out‐of‐plane asymmetry. The CD arises from the strong near‐field coupling plasmonic resonation. The resonant intensities vary for right or left circular polarization, leading to different absorptions. It is noted that local optical chirality is higher for right circular polarization than for left one. The total chirality is calculated, and they found the strongest chirality at the edges of the antenna and an integrated chirality value 5 times that of Au. The THz chiral metasurface highly enhances the sensitivity of chirality detection. The detection of bovine serum albumin concentrations revealed that the protein molecules accumulate close to the antennas for smaller concentrations, giving an intense signal. For higher concentrations, the saturation of this device leads to the detection of the signal provided by molecules far from the antennas. The achieved sensing range is 0.5–50 mg mL^−1^. A further example of SERS applied to metasurfaces with random‐oriented metallic NPs functionalized with aptamers is represented by the work of Bhamidipati et al.^[^ ^42^ ^]^ Au nanostars (AuNSs) were deposited on an APTES‐coated glass substrate and functionalized with the aptamer for the cellular tumor marker EpCAM. Using 4‐aminothiophenol (4‐ATP) as Raman reporter and 6‐mercaptohexanol (MCH) to prevent unspecific bindings, the activated substrate obtained successfully detected EpCAM, both soluble and cell membrane‐embedded at single cell level (Figure 7d).^[^ ^42^ ^]^ The functionalized device could also exploit nucleic acid sequences for biosensing via Watson‐Crick hybridization. Indeed, a glass substrate coated with AuNPs was exploited by Ge et al. to recognize CpG methyltransferase (M.SssI) activity. M.SssI is an enzyme capable of binding to DNA and causing methylation, often associated with cancer. The methylated DNA from the serum of cancer patients was appropriately amplified by rolling circle amplification (RCA). The single strands obtained from RCA were then hybridized with a complementary sequence tagged with the Raman reporter 5‐carboxyfluorescein (5‐FAM) linked to AuNPs. The activity of the M.SssI enzyme was proportional to the signals obtained by SERS.^[^ ^43^ ^]^ Li et al. created a metasurface to detect the tumor marker Tyrosinase, whose activity is associated with melanoma, in the serum of various patients. AuNRs were deposited on a glass substrate and functionalized with dopamine via the linker di(N‐succinimidyl)‐3,3′‐dithiodipropionate (DSP). 4‐mercaptophenylboronic acid (4‐MPBA)‐coated AgNPs were added as SERS probes. In the presence of Tyrosinase, dopamine is oxidized to dopaquinone, causing a decrease in the SERS signal proportional to the amount of Tyrosinase (Figure 7e).^[^ ^44^ ^]^

Incorporating plasmonic metamaterials in flexible substrates can provide new functionalities and integrated applications. These devices can be stretched, bent, or deformed into different shapes, overcoming rigid platform restrictions. The demand for such versatile and multifunctional compounds mainly comes from biomedical applications to guarantee the integration of sensors in the human body or for in vivo biological sensing. Bottom–up techniques are necessary to enhance the scalability of these products and permit their application in real life. A further example is the glucose‐sensor provided by Y. Ziai et al. in this case, the unique properties of the chameleon skin inspired the realization of the flexible sensor.^[^ ^45^ ^]^ Two layers characterize the chameleon skin; the upper is responsible for skin coloration, while the lower provides thermoregulation. The guanine nanocrystals of the upper layer constitute a high *n* material arranged on a low *n* material, namely the cytoplasm. Similarly to photonic crystals, they produce a lattice‐like structure. Likewise, the proposed biosensor exploits light–matter interaction without requiring external stimuli. The associated plasmonic device comprises two external layers of plasmonic hydrogel (AgNCs embedded in the hydrogel matrix characterized by SEM and atomic force microscopy) separated by the layer of electrospun mat. In addition to the sensing features due to AgNPs, the system exhibits excellent photo‐thermal and antibacterial properties. The calibration curve of the experimental plasmonic shift as a function of the glucose concentration revealed the LOD value of 2.29 mm. The device was validated for D‐glucose analysis within a wide range of concentrations. The antibacterial activity was studied by seeding the *S. aureus* solution on the sample surface. After 4 h of incubation, the hydrogel containing AgNPs eliminated 99.9% of bacteria, while the same starting colonies were recovered from the bare hydrogel. Moreover, introducing the nanofibrous mat results in outstanding mechanical properties: the final system shows excellent flexibility and can be folded and bent without structural damage. However, a compromise between scalability and sensitivity is necessary in some cases. X. Liu et al. constructed a flexible plasmonic nanostructure combining a bottom–up self‐assembly step with a top–down second one.^[^ ^46^ ^]^ The first step was carried out to create nanoscale features through the Langmuir‐Blodgett technique and fabricate a layer of closely‐packed polystyrene (PS) beads on a silica wafer, while the second was to realize the desired pattern through laser engraving. In the end, adhesive tape enabled the template's transfer and the metastructure's realization. An Ag film was deposited on the tape, obtaining the flexible plasmonic metafilm. The strongest SERS effect was observable under a 633 nm laser source using 540 nm PS beads and 280 nm Ag film. Compared to other devices, the advantage was ascribable to the possibility of quantitatively measuring analyte concentration on non‐planar surfaces and thus integrating the metasurface into devices, thanks to its mechanical flexibility. Indeed, the authors used the metasurface to realize a sensing vial to be mounted on an enzyme‐linked immunosorbent assay (ELISA). The vial can be integrated into any ELISA as a secondary accessory to improve the analysis of liquid samples in point‐of‐care tests. An additional device for the point‐of‐care analysis of liquid samples was obtained by integrating the metasurface in a flow cell, allowing dynamic Raman analysis of analytes. The detection limit was tested using the organic dye crystal violet and was found to be 1 nm for both devices. As a final application, the authors realized a proof‐of‐concept of a wearable multifunctional medical 1D bracelet for collecting safety information that may alert the medical staff. The bracelet can recognize various analytes with several SERS sensing units. In addition, it is possible to store the wearer's health data in it, encrypting them in a QR code thanks to the optical properties of the metasurface. This provides additional useful functionality in the healthcare world, allowing medical personnel to obtain information about the patient without violating privacy.^[^ ^46^ ^]^

## 5 Photo‐Thermal Applications of Next‐Generation Bottom–Up Metasurfaces

**Figure 8:**
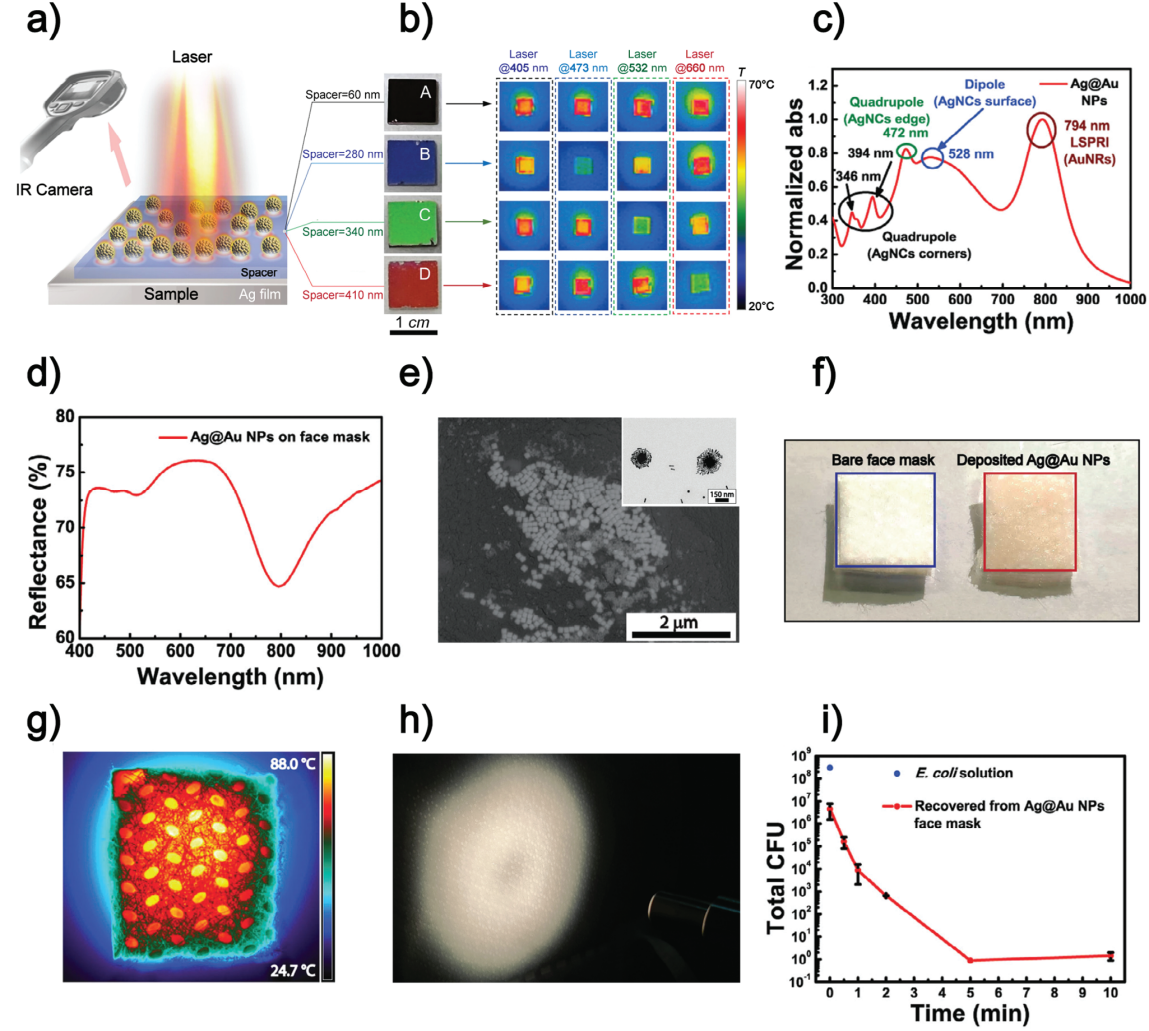
Self‐assembled optical metasurfaces applied in the biomedical field. a) On the left is the IR thermal imaging schematic of the disordered bottom‐up metasurface under a laser source. On the right are photographs of the four metasurfaces fabricated with a dielectric spacer with 60, 280, 340, and 410 nm of thickness. b) Thermal images of the metasurfaces irradiated under 405, 473, 532, and 633 nm laser sources. Reproduced with permission.^[^ ^55^ ^]^ 2023, American Chemical Society. c) Optical absorption spectroscopy of the hybrid heterostructure. d) Diffuse reflectance spectroscopy of the functionalized FFP2 face mask fibers. e) SEM micrograph of the functionalized FFP2 face mask fibers. (Inset) Transmission electron microscopy (TEM) micrograph of the heterostructures composed of AgNCs surrounded by AuNRs. f) Photo of the bare face mask on the left and of the reddish functionalized face mask on the right. g) High‐resolution thermal image and h) photo of the functionalized FFP2 face mask irradiated by the white light source. i) Colony counting of the bacterial cells after different irradiation time intervals. Reproduced with permission.^[^ ^37^ ^]^ 2024, Wiley‐VCH GmbH.

Metal NPs are largely exploited as photo‐thermal agents to overcome the limitations of organic photosensitizing drugs. Plasmonic NPs present high biocompatibility and enhanced absorption cross‐section, making minimally invasive therapy and high photostability possible in biomedical applications without photobleaching effects.^[^ ^47^ ^]^ The outstanding properties of the noble metal NPs rise from the LSPR phenomenon that occurs when the resonant wavelengths of the light source irradiate them. The absorption of the incident electromagnetic radiation is confined and enhanced within the boundaries of the NPs. The resulting collective oscillations of free electrons on the surface of NPs increase the frequency of their collisions with the lattice atoms, generating the Joule heating. The thermal energy is then released to the surrounding environment. This metal NP's capability to convert light into heat is called the thermoplasmonic effect.^[^ ^29^ ^]^ The material composition, size, shape, and surrounding medium of the metal NP strongly affect the density of the electrons on the NP surface, tuning its optical and photo‐thermal response.^[^ ^48^ ^]^ The versatility of these colloidal dispersions makes them ideal candidates for many biomedical applications such as biosensing, disinfection, drug delivery, and therapy.^[^ ^49^, ^50^, ^51^, ^52^ ^]^ The arrangement of plasmonic NPs as meta‐atoms in metasurfaces on rigid or flexible substrates further widens the possible uses.^[^ ^53^, ^54^ ^]^ JJ. Chen et al. investigated experimentally and theoretically the photo‐thermal response of random and bottom‐up metasurfaces, tuning their optical properties.^[^ ^55^ ^]^ First, an Ag thin film was deposited on a silica wafer through thermal evaporation, and then a dielectric spacer (lithium fluoride or dialuminium trioxide) was grown. After that, Au nanoclusters were produced on this surface through the gas‐phase cluster beam technique, realizing a disordered arrangement of the nanostructures. By tailoring the dielectric spacer thickness from 40 to 450 nm, the wavelength absorption can be tuned in the visible range toward higher wavelengths, and the photo‐thermal conversion efficiency is accordingly controlled. Each sample was irradiated under 405, 473, 532, and 660 nm continuous wave  lasers with a power density of 100 mW cm^−2^. **Figure** **8**a shows the schematized black sample corresponding to the broadband regime given by the thinner spacer. In contrast, the blue, green, and red samples are associated with defined absorption wavelengths in the visible range. As a result, in Figure 8b, the samples are heated only if the laser wavelength matches the absorption band of the metasurfaces. Disordered plasmonic metasurfaces can thus achieve both broadband and wavelength‐selective absorption, varying the dielectric spacer thickness.

G. Palermo et al. demonstrated that the photo‐thermal properties of a plasmonic flexible substrate can be controlled by exploiting the mechanical properties of the elastomeric tape.^[^ ^56^ ^]^ The inter‐particle distance's mechanical control is used to enhance the heat generation reproducibly. A 33% temperature increase is reached upon stretching at the 250 mW power of the CW pump green laser. On the contrary, Q. Zou et al. exploited the photo‐thermal properties of a metasurface consisting of Au nanostructures deposited on PDMS to vary its morphology and, consequently, its optical features.^[^ ^57^ ^]^ The applied current induced the heating under a total power consumption of 10.5 mW through the Joule effect, and it produced an expansion of the PDMS that caused the 39 nm blue‐shift of the plasmonic peak. The study and realization of photo‐responsive flexible materials functionalized with plasmonic NPs pave the way for innovative approaches in the biomedical field. As an example, nanotechnology was a powerful tool for facing the drawbacks of the high demand for personalized protective devices during the COVID‐19 pandemic, which increased the production of face masks and raised environmental pollution. To overcome the difficulties of daily use of face masks, which are difficult to dispose of, we obtained paradigm change by realizing a multifunctional FFP2 face mask that can be disinfected if irradiated under a conventional white light source and can selectively recognize the interacting pathogens with the addition of a highly sensitive optical metasurface.^[^ ^37^ ^]^ We realized a plasmonic broadband light absorber, the self‐assembled Ag@Au NPs hybrid heterostructures, composed of AgNCs surrounded by AuNRs for improved photo‐thermal efficiency under white light irradiation. Their absorption spectrum reveals that the collected frequencies range from the Visible to the NIR range (Figure 8c), maintaining its constituents' optical fingerprints. The hydrophobic FFP2 surface was lowered through oxygen plasma treatment, and the hybrid heterostructures were deposited by drop casting. Still, the optical absorbance plasmon peaks are visible from the diffuse reflectance spectroscopy of the functionalized face mask but with a stronger AuNRs signal (Figure 8d) due to their high coverage of the AgNCs, as the SEM micrographs reveal (Figure 8e). Figure 8e insert of the TEM micrograph of the colloidal dispersion shows the core@shell geometry of the Ag@Au NPs defined by the AuNRs surrounding the AgNCs: this structure remains stable even when deposited on the FFP2 fibers. The deposition through drop casting on the activated FFP2 face mask fibers confers the slight reddish appearance of the FFP2 fibers (insert in Figure 8f). The broad range collected by these hybrid heterostructures enables the high photo‐thermal efficiency of the device under white light irradiation to be 68.8%. The high‐temperature values achieved by the functionalized face mask under white light irradiation and collected by a high‐resolution thermal camera are close to 90 °C (Figure 8g). This value becomes even higher when the samples were contaminated with *E. coli* bacterial solutions (+9 °C). The photo‐thermal disinfection was performed, irradiating the medical device's surface with an optical fiber covering a large area of the face mask surface (Figure 8h) without affecting its protective function but confirming the possibility of being reused. The *E. coli* inactivation of well above 6 logs (Figure 8i) highly exceeds the result of Annesi et al., who reported a 2 log reduction in bacterial viability using AuNPs under resonant laser light and is close to the results reported by Rowan et al., where high‐intensity UV pulses were used as disinfection treatment but did not enable the same result on different strains that may not be affected by UV light: gram‐negative bacteria are more sensitive to the effects of UV light, and thus the same result is not achieved on different strains.^[^ ^50^, ^58^ ^]^ The disinfection mechanism given by the metallic heterostructures deposited on a dielectric layer of the face mask fibers is then combined with the detection capabilities of an optical metasurface composed by an array of AgNCs assembled on an Au thin layer through the eLbL bottom‐up method.^[^ ^17^ ^]^ As discussed in Section 3, the optical metasurface‐based biosensor exhibited an impressive LOD (1 CFU in 100 mL) that largely surpassed the one of other self‐assembled metastructures used in biomedical sensing.^[^ ^59^ ^]^ The easy and accessible disinfection of face masks is primarily intended for use in hospitals and clinical settings, as well as in field hospitals where there is an urgent need for a convenient and safe light source. For this purpose, both a standard white light source, such as a smartphone flashlight, and renewable solar energy can be effectively utilized. Approximately 42–43% of the solar spectrum consists of visible light. Current experiments are focused on harnessing the photo‐thermal response of plasmonic heterostructures under solar light while also assessing the additional benefits of NIR wavelengths, which are present in the absorption spectrum of the heterostructures but are not utilized when illuminated with a white light source.

## 6 Conclusions and Future Perspectives

Bottom‐up metasurfaces are a new generation of optical components realized by employing more affordable fabrication techniques such as self‐assembly of metallic subunits (meta‐atoms).

**Table** **1** also points out that the working spectral range of the bottom–up hybrid metasurface encompasses the visible and NIR wavelengths. However, some studies demonstrate the possibility of fabricating metasurfaces that are also active in the THz range. The bottom‐up approach also allows using several materials, including metals, dielectrics, and hybrid and flexible materials. Au nanocrystals are widely utilized as plasmonic resonators. However, other organic materials, such as carbon bars and polystyrene beads, demonstrate attractive opportunities.

**Table 1:** Summary table of hybrid metasurfaces fabricated by bottom‐up or lithography‐free processes along with the respective possible applications.

Fabrication technique	Meta‐atom	Spectral range	Application(s)	Ref.
eLbL	AgNCs	600–1600 nm	Active devices	[16]
Resistive thermal evaporation	70 nm thick Au layer	500–800 nm	Sensing	[40]
Laser graphene technology	Twisted stacking carbon bars	0.4–1 THz	Biosensing	[41]
Au‐APTES binding	Au nanostars	600–2000 cm^−1^	Biosensing	[42]
Langmuir‐Blodgett + self‐assembly	Au@SiO_2_	1100–1800 cm^−1^	Biosensing	[43]
Marangoni effect	AuNRs	400–1800 cm^−1^	Biosensing	[44]
Langmuir–Blodgett, laser engraving, stick and peel	Polystyrene beads	1000–1800 cm^−1^	Sensing and encryption	[46]
Hydrogel matrix	AgNCs	350–800 nm	Biosensing	[45]
Dipping	AuNPs	460–580 nm	Opto‐mechanical temperature control	[56]
Metal‐assisted transfer	Au wires	900–1600 nm	Flexible thermo‐mechanical modulator	[57]
Drop casting + LbL	Ag@Au heterostructures + AgNCs	300–1000 nm	Photo‐thermal disinfection + sensing	[37]
LbL	AgNCs	300–1000 nm	Light‐controllable optical components	[17]
Self‐assembly (covalent)	AuNPs	400–700 nm	Biosensing	[32]
Self‐assembly (covalent)	AuNPs	/	Electrochemical biosensing	[36]
eLbL	AuNRs	400–1100 nm	Biosensing	[31]
eLbL	AuNRs	400–1100 nm	Disinfection	[29]
eLbL	AuNRs	400–1100 nm	Biosensing and disinfection	[27]
Self‐assembly	AuNPs + AgNPs	250–800 nm	Biosensing	[33]
Self‐assembly (covalent)	Au nanoflowers	400–900 nm	Biosensing	[35]
Thermal dewetting	Au nanoislands	644–651 nm	Biosensing	[39]
Self‐assembly	AgNCs	200–2600 nm	Tunable optical components	[18]
Electrodeposition of PANI	AuNPs	400–700 nm	Electrically tunable dichroic multicolor nanofilter	[20]
Spin coating + EBL	AuNCs functionalized with DNA	1000–4000 nm	Catalysis, telecommunications, and quantum computing	[21]
LbL dip coating + PECVD	AgNCs, Ag cuboctahedra, Au/Ag/Pt nanospheres silica–Au core–shell nanospheres	330–2740 nm	Spectrally tunable photodetectors or beam‐steering surfaces	[22]
Bonding method	AuNR trimers	500–1000 nm	Sensing, display, and communication	[23]
EBL + spin coating	AuNCs octahedral NPs, decahedral NPs, cuboctahedral NPs and concave dodecahedral NPs	610–690 nm	Dynamically tunable anomalous reflector; flat lenses, optical cloaking devices, and holograms	[24]

Because of the realization techniques, bottom‐up metasurfaces are easily scalable, allowing for large‐area production, and are generally more cost‐effective than top–down methods. This approach enables the realization of sophisticated designs at the nanoscale, offering excellent complexity and precision. Undoubtedly, the next challenge in bottom–up metasurface fabrication is the realization of ordered metasurfaces via bottom–up techniques. Currently, assembly techniques allow the meta‐atom organization in an a‐priori‐designed arrangement, only with short‐range order. From a mid‐term perspective, exploring and deeply investigating hybrid fabrication techniques will be necessary. As for a few examples in this review, hybrid fabrication techniques would merge the exploitation of a template with the utilization of colloidal techniques, resulting in ordered bottom–up metasurface prompts for the scaling‐up. The upcoming outlook for ordered bottom‐up metasurface fabrication can also include using the 3D printing techniques as a further hybrid fabrication approach.

The development of scalable bottom–up ordered metasurfaces is a crucial issue, as they are adaptable to emerging materials like 2D materials and biomaterials, making them valuable for advanced applications in optics, sensors, and energy technologies. In the present work, we have highlighted the extraordinary progress researchers have performed in the past years for boosting innovative biomedical applications that rely on the exotic properties of metasurfaces. In particular, as evidenced in Table 1, the random organization of plasmonic nanomaterials has been crucial for realizing innovative applications such as biosensors for detecting pathogens or biomolecules. The extraordinary thermo‐optical properties of bottom–up metasurfaces have been key to performing new‐generation treatments for thermal disinfection and stimuli‐responsive biomedical applications. Integrating metasurfaces and smart materials could represent a new paradigm for realizing innovative applications in the future.

For instance, the possibility to dynamically tune their optical properties opens unique applicative opportunities ranging from advanced optical components to quantum computing. Artificial intelligence (AI) can offer promising support in enhancing metasurface fabrication and applications. Specialized algorithms can be employed to select the most effective sub‐wavelength elements to be used as meta‐atoms, tailored to the target analyte and optimized for the appropriate wavelength range. Additionally, AI can help determine the most effective combinations of meta‐atoms and probe molecules. Furthermore, AI can identify and analyze output signals related to side phenomena associated with biorecognition events, leading to a broader range of analytical results. Finally, thanks to the utilization of AI, bottom‐up hybrid metasurfaces can be used in modern healthcare facilities to realize early diagnosis kits for medical purposes or remotely controlled sensors to monitor patients' health.

## Conflict of Interest

The authors declare no conflict of interest.
